# H-IPSE Is a Pathogen-Secreted Host Nucleus-Infiltrating Protein (Infiltrin) Expressed Exclusively by the Schistosoma haematobium Egg Stage

**DOI:** 10.1128/IAI.00301-17

**Published:** 2017-11-17

**Authors:** Luke F. Pennington, Abdulaziz Alouffi, Evaristus C. Mbanefo, Debalina Ray, David M. Heery, Theodore S. Jardetzky, Michael H. Hsieh, Franco H. Falcone

**Affiliations:** a Stanford University School of Medicine, Stanford, California, USA; b School of Pharmacy, Division of Molecular Therapeutics and Formulation, University of Nottingham, Nottingham, United Kingdom; c Biomedical Research Institute, Rockville, Maryland, USA; d Department of Pathology, University of California, San Francisco, California, USA; e School of Pharmacy, Division of Biomolecular Science and Medicinal Chemistry, University of Nottingham, Nottingham, United Kingdom; f Department of Urology, The George Washington University, Washington, DC, USA; Cornell University

**Keywords:** Schistosoma haematobium, nuclear localization signal, schistosomiasis

## Abstract

Urogenital schistosomiasis, caused by the parasitic trematode Schistosoma haematobium, affects over 112 million people worldwide. As with Schistosoma mansoni infections, the pathology of urogenital schistosomiasis is related mainly to the egg stage, which induces granulomatous inflammation of affected tissues. Schistosoma eggs and their secretions have been studied extensively for the related organism S. mansoni, which is more amenable to laboratory studies. Indeed, we have shown that IPSE/alpha-1 (here M-IPSE), a major protein secreted from S. mansoni eggs, can infiltrate host cells. Although the function of M-IPSE is unknown, its ability to translocate to the nuclei of host cells and bind DNA suggests a possible role in immune modulation of host cell tissues. Whether IPSE homologs are expressed in other schistosome species has not been investigated. Here, we describe the cloning of two paralog genes, H03-IPSE and H06-IPSE, which are orthologs of M-IPSE, from egg cDNA of S. haematobium. Using PCR and immunodetection, we confirmed that the expression of these genes is restricted to the egg stage and female adult worms, while the H-IPSE protein is detectable only in mature eggs and not adults. We show that both H03-IPSE and H06-IPSE proteins can infiltrate HTB-9 bladder cells when added exogenously to culture medium. Monopartite C-terminal nuclear localization sequence (NLS) motifs conserved in H03-IPSE, SKRRRKY, and H06-IPSE SKRGRKY, are responsible for targeting the proteins to the nucleus of HTB-9 cells, as demonstrated by site-directed mutagenesis and green fluorescent protein (GFP) tagging. Thus, S. haematobium eggs express IPSE homologs that appear to perform similar functions in infiltrating host cells.

## INTRODUCTION

Schistosomes are digenetic blood trematodes, which rely on their egg stage for transmission to the intermediate host, a water snail (1). In order to reach the aquatic environment, the eggs deposited by adult female worms in the blood vessels of their mammalian host have to cross several layers of host tissue before they can reach the lumen of the gut or, in the case of Schistosoma haematobium, the bladder. This is a critical step in the life cycle of the parasite and is therefore very likely to have been fine-tuned to the host's immunological and tissue environment during the course of evolution. The microenvironments of the bladder and the gut are histologically and immunologically quite different, and this may be reflected in differences between the molecules produced by the eggs of S. haematobium and Schistosoma mansoni and the underlying mechanisms leading to the translocation of eggs across tissues. Most proteomic studies have concentrated on the egg stage of the more available trematode S. mansoni (2–4); those studies identified three major protein components (3) produced by mature eggs: omega-1 (5), kappa-5 (6), and IPSE/alpha-1 (7).

Here, we show that S. haematobium expresses multiple variants of a protein homologous to IPSE/alpha-1 in S. mansoni (M-IPSE), which we have called H-IPSE, a term that is used here to collectively describe the different orthologs of M-IPSE in S. haematobium. The mRNA expression of H-IPSE is restricted to the egg and female worm stages but translated as protein only in eggs. H-IPSE shares an important biological activity described for M-IPSE: the ability to be taken up by and translocate to the nucleus of host cells.

## RESULTS

### Identification of H-IPSE variants.

**FIG 1:**
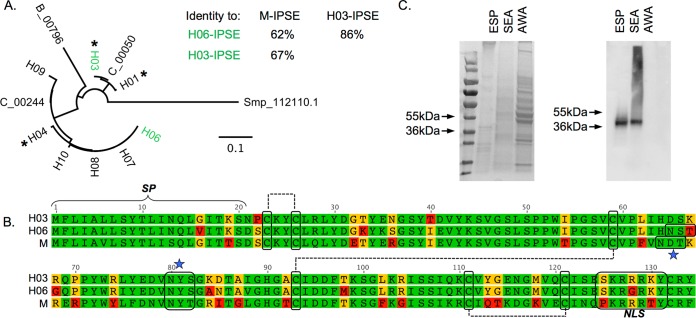
S. haematobium expresses multiple forms of H-IPSE. (A) Amino acid sequences of predicted H-IPSE paralogs, M-IPSE, and sequenced transcripts from egg cDNA were globally aligned by using a Blosum62 cost matrix, and a tree was built by using the neighbor-joining method in Geneious 7.1.4. The bar represents amino acid substitutions per site. H06 and H03 variants chosen for expression are highlighted in green, and their respective amino acid identities and identities to M-IPSE are shown. All variants identified through 3′-RACE cloning are indicated with asterisks. (B) Alignment of amino acid sequences of H03-IPSE (top rows) and H06-IPSE (middle row)s with the sequence of the homolog in S. mansoni (IPSE/alpha-1; here named M-IPSE) (bottom rows). These H-IPSE clones retain ∼63 to 68% amino acid identity and several features described previously for M-IPSE (24), including a 20-amino-acid classical signal sequence, seven cysteine residues involved in disulfide bonds, two N-linked glycosylation consensus motifs, and a predicted nuclear localization sequence. Residues shown in green are identical, residues in yellow share properties (e.g., hydrophobicity or polarity), and residues in red lack similarity. (C) SDS-PAGE gel (left) and Western blot with anti-H06-IPSE antiserum (right). Lanes contain parasite-derived adult worm antigen (AWA), egg secretory protein (ESP), or soluble egg antigen (SEA).

To identify homologs of S. mansoni IPSE in the S. haematobium genome, we performed a BLAST search at WormBase ParaSite (http://parasite.wormbase.org) using the predicted transcript of M-IPSE (Smp_112110.1) (8–10). This analysis identified three paralogs within the S. haematobium genome, all with predicted transcripts with high identity to M-IPSE (C_00050 [67% amino acid identity], C_00244 [63%], and B_00796 [56%]). To verify these transcripts, we isolated cDNA from S. haematobium eggs and employed two strategies. The first strategy employed 5′ and 3′ primers designed to amplify transcripts predicted from the C_00244 locus, and the second strategy employed a 3′-rapid amplification of cDNA ends (RACE) cloning strategy with a 5′ primer targeting highly conserved regions of all H-IPSE variants (see Table S1 in the supplemental material). In total, 14 IPSE transcript sequences were obtained, and 8 Sanger sequencing runs contained data sufficient for unambiguous base calling throughout the open reading frames (ORFs). These transcripts clustered with two of the three H-IPSE paralogs (Fig. 1A), and one transcript from each cluster (H03 and H06) was selected for further study. Sequence variations within these clones suggest that H-IPSE genes are polymorphic (Fig. S1 to S3).

An alignment of H03-IPSE/H06-IPSE with M-IPSE (Fig. 1B) demonstrates the conservation of the seven cysteines, known to form three intramolecular disulfide bonds and one intermolecular bond, resulting in a homodimeric structure. Two potential N-linked glycosylation consensus motifs with small variations are also present. A 20-amino-acid-long N-terminal classical secretory sequence (CSS) is predicted for both H03- and H06-IPSE by SignalP 4.1 (11). To verify the presence of the IPSE protein in S. haematobium parasite-derived material, we expressed and refolded H06-IPSE from insoluble inclusion bodies and used bacterially derived H06-IPSE to generate polyclonal anti-H-IPSE antibodies in rabbits (Fig. S1). On Western blots, anti-H-IPSE antibodies bound an ∼40-kDa protein species in both S. haematobium egg secreted protein and soluble egg antigen but not in adult worm extracts (Fig. 1C). This corresponds to the expected size for dimeric, glycosylated H-IPSE variants.

### H03- and H06-IPSE variants have a predicted nuclear localization sequence.

Several algorithms (cNLSMapper [12], PSORT II [13], NLStradamus [14], and NucPred [15]) predict a C-terminal nuclear localization sequence (NLS) in H03-IPSE close to the C terminus (data not shown), similarly to the NLS described previously for M-IPSE (16). Intriguingly, the H06-IPSE paralog carried an R128G variant within the nuclear localization sequence corresponding to the validated NLS (16) in M-IPSE (Fig. 1B). Such variants have also been observed in S. mansoni studies. For example, ESP3-6, a protein later recognized as an M-IPSE variant, is 97% homologous to the reported M-IPSE sequence (GenBank accession no. AAK26170.1) but contains an R132L variant within the NLS (ESP3-6 GenBank accession no. AF527011). Positively charged amino acids in an NLS are key to its nuclear targeting activity mediated by binding to cytosolic importin-α (17); thus, such a replacement will have an impact on the protein's ability to translocate to the nucleus, ranging from less efficient translocation to no translocation at all, depending on the exact position in the NLS (17). Substitutions in the NLS will potentially also have an effect on DNA binding specificity. This is also reflected in the less certain prediction of an NLS in H06-IPSE by cNLSMapper and the other tested algorithms (data not shown).

### The NLS in H03- and H06-IPSE is able to direct a large fluorescent protein to the nucleus of mammalian cells.

**FIG 2:**
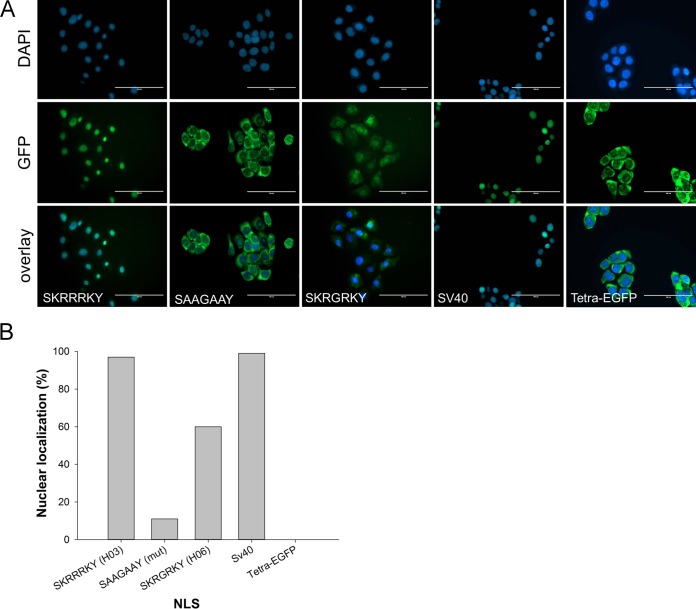
Effect of multiple amino acid substitutions on the NLS in H-IPSE. (A) The nucleotides encoding the H03/H06-IPSE nuclear localization sequence (SKRRRKY and SKRGRKY, respectively) were inserted into the pTetra-EGFP construct (2, 3). pTetra-EGFP encodes a tetrameric EGFP construct resulting in the expression of a fluorescent protein, which, due to its size (>100 kDa), is excluded from the nucleus in the absence of a functional NLS (tetra-EGFP) or imported into the nucleus in the presence of a functional NLS (canonical SV40 NLS and H03/H06-IPSE NLS). Nuclei were stained with DAPI, and green fluorescence was measured with the GFP light cube on an Evos fl microscope, 24 h after transfection. Bar, 100 μm. (B) Comparison of the effects of wild-type H06-IPSE and H03-IPSE and mutant H03-IPSE NLSs on the nuclear localization of the tetra-EGFP fusion protein. One hundred transfected HTB9 cells were evaluated under an Evos fl microscope for each transfection, and the percentage of cells displaying exclusively nuclear fluorescence, as opposed to cytosolic localization only or mixed cytosolic/nuclear localization, was recorded. The positive control was the SV40 canonical NLS, and the negative control was the unmodified tetra-EGFP vector (Tetra-EGFP).

Therefore, in order to assess functionality of the predicted NLS in H03-IPSE and the potential impact of the R128G substitution in H06-IPSE, we cloned oligonucleotides encoding the predicted NLSs into the previously described tetra-enhanced green fluorescent protein (EGFP) vector (16). The resulting constructs were then transfected into HTB-9 uroepithelial carcinoma cells as a model of host cells relevant for S. haematobium infection. The results of the transfection of the tetra-EGFP vector encoding the NLSs of H03-IPSE (SKRRRKY) and H06-IPSE (SKRGRKY), as well as the predicted mutant NLS SAAGAAY (Fig. 2), confirm that the H03-IPSE NLS is fully functional, resulting in the complete translocation of the large tetra-EGFP protein into the nucleus. In the H06-IPSE NLS, the presence of an uncharged G residue in the charged KRRRK H03-IPSE core sequence of the NLS appears to weaken its strength as a nuclear targeting signal, as documented by the mixed cytosolic/nuclear localization, in contrast to the exclusively nuclear localization with the H03-IPSE NLS SKRRRKY (Fig. 2B). This difference is consistent with the results obtained with different prediction algorithms (not shown). The replacement of lysine and arginine with alanine (SAAGAAY) results in a nonfunctional NLS, which is no longer able to translocate the tetra-EGFP protein into the nucleus (Fig. 2A and B). These results clearly show that while the predicted NLS in H03-IPSE is fully functional, the G substitution in the positively charged core compromises this function at least in part. Further substitutions almost fully ablate NLS functionality.

### Recombinant M-IPSE and H-IPSE can be expressed at high yields in HEK293-6E cells grown in suspension.

**FIG 3:**
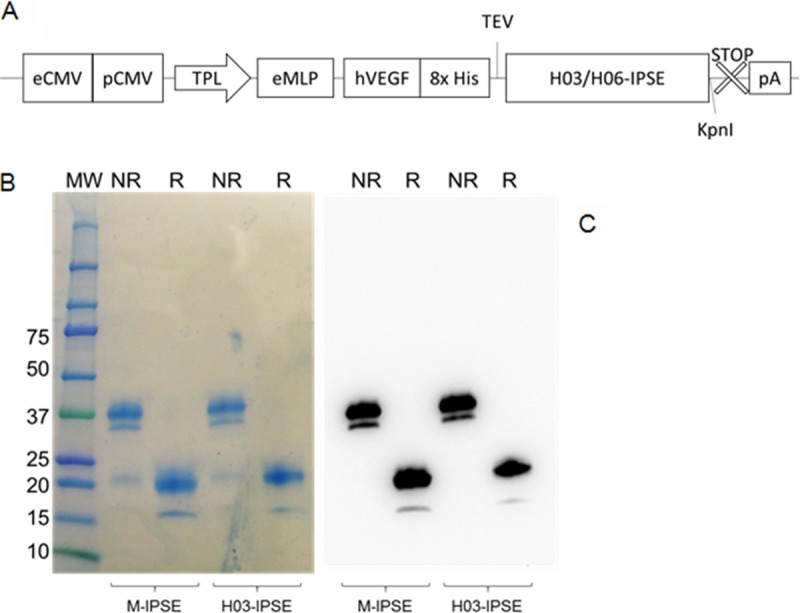
Expression of M-IPSE and H-IPSE in HEK293-6A cells. (A) Schematic diagram of the pTT5 H03/06-IPSE expression cassette. eCMV, cytomegalovirus enhancer sequence; pCMV, cytomegalovirus promoter; TPL, tripartite leader sequence from adenovirus; eMLP, enhancer element from the adenovirus major late promoter; hVEGF, human vascular endothelial growth factor signal sequence; 8× His, octahistidine tag; TEV, tobacco etch virus protease cleavage site; STOP, stop codon; pA, β-globin polyadenylation signal. (B and C) Coomassie-stained 4-to-20% gradient SDS-PAGE gel (B) and Western blotting (C) of recombinant H03-IPSE (and M-IPSE, used for comparison) expressed in HEK293SF-3F6 cells, purified by IMAC from the serum-free culture supernatant, and run under nonreducing (NR) or reducing (R) conditions. MW, molecular weight marker (in thousands).

For subsequent experiments, we cloned and expressed H-IPSE using HEK293-6E cells. This system uses a serum-free-medium-adapted clone and allows HEK293 cell culture in suspension, enabling high cell densities and recombinant protein yields. Proteins generated by using these cells are glycosylated, which more closely parallels the glycosylation of native proteins. Recombinant protein can be harvested from culture supernatants after transient transfection and can be purified, e.g., via immobilized-metal affinity chromatography (IMAC) using an 8× His tag in the construct (Fig. 3A).

The results shown in Fig. 3B demonstrate the successful expression of H03-IPSE as a mostly dimeric protein with a molecular mass of approximately 38 to 40 kDa, with small amounts of monomeric protein of about 20 kDa, in line with what we described previously for M-IPSE (18, 24). The double bands are presumably due to glycosylation variants, which are well described for M-IPSE (18). The purity in eluted fractions after IMAC was high and did not require any additional purification steps for downstream experiments. In our hands, IPSE proteins produced a range of yields, with H03-IPSE exhibiting the lowest final yield (∼5 to 10 mg/liter) and M-IPSE and the H06-IPSE SKAAAKY NLS mutant producing the highest yield (∼15 to 25 mg/liter) (see Fig. S5 in the supplemental material). Attempts to concentrate the protein to a higher concentration above 0.5 mg/ml resulted in the formation of aggregates, which was not seen by SDS analysis but appeared in size exclusion chromatography experiments.

### Recombinant H03-IPSE added exogenously is taken up by HTB-9 uroepithelial cells and efficiently translocates to the nucleus.

**FIG 4:**
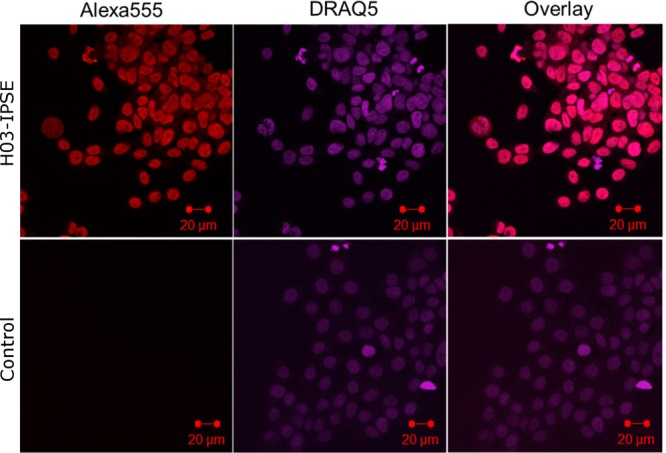
Recombinant H03-IPSE is taken up by HTB-9 host cells and translocates to the nucleus. HTB-9 cells, incubated for 24 h with 0.40 nM recombinant H03-IPSE, were stained with 5 μM DRAQ5 nuclear stain for 15 min at room temperature, followed by staining with a mouse anti-His antibody and Alexa Fluor 555-conjugated goat anti-mouse IgG(H+L) as a secondary antibody. The right column shows the overlay of the two channels. Uptake in HTB-9 cells was visualized by confocal microscopy. The primary anti-His antibody was omitted in the control lane.

To mimic S. haematobium infection conditions, we added recombinant 8× His-tagged H03-IPSE to the culture medium of proliferating HTB-9 uroepithelial carcinoma cells as described in Materials and Methods. After 24 h, fixed cells were immunostained with an anti-His tag antibody. As shown in Fig. 4, this revealed the highly efficient uptake of exogenous H03-IPSE, which was present in HTB-9 nuclei, as revealed by costaining of nuclear DNA with DRAQ5 (Thermo Fisher Scientific). The nuclear staining pattern suggests that H03-IPSE was largely excluded from the nucleolar regions. This result indicates that the H03-IPSE protein can infiltrate the vast majority of cells with a remarkable efficiency and localize to the nucleus. The only cells that we observed that did not stain for recombinant H03-IPSE were those actively undergoing mitosis, in which the nuclear membrane is broken down.

### The NLS in H03- and H06-IPSE is essential for nuclear translocation but not for cellular uptake.

**FIG 5:**
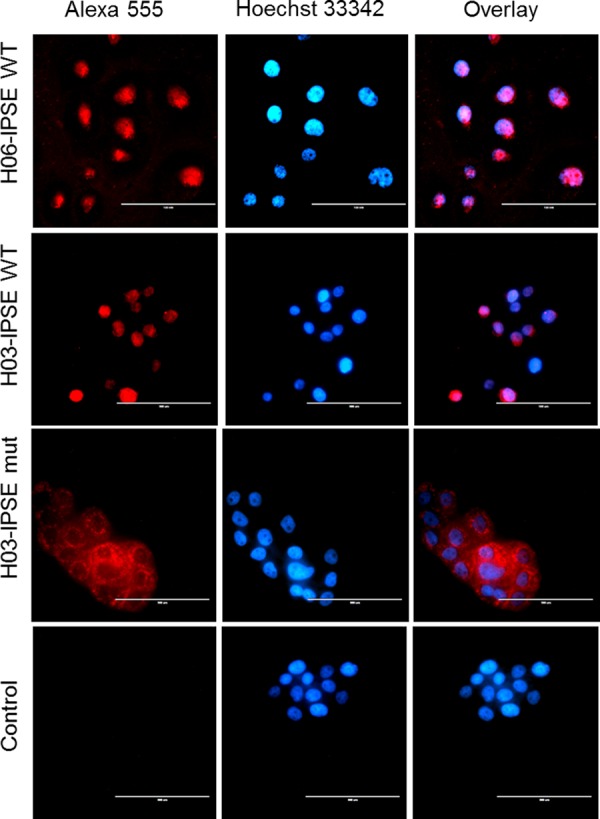
Fluorescence microscopy of HTB-9 cells incubated with recombinant H03-IPSE (NLS, SKRRRKY), H06-IPSE (NLS, SKRGRKY), or the H03-IPSE mutant (NLS, SKAAAKY). HTB-9 cells were stained with Hoechst 33342 nuclear stain for 15 min at room temperature, followed by staining with a mouse anti-His antibody and Alexa Fluor 555-conjugated goat anti-mouse IgG(H+L) as a secondary antibody. The right column shows the overlay of the two channels. The primary anti-His antibody was omitted in the control lane. Bar, 100 μm.

Next, we compared the abilities of both H-IPSE variants and the NLS alanine mutant to gain access to the nuclear compartment of HTB-9 cells when added to cell culture medium. The results, visualized with a fluorescence microscope, are shown in Fig. 5. When the subcellular localization of the recombinant molecules with a molecular mass of approximately 40 kDa was assessed, both the H03- and H06-IPSE variants seemed similarly efficient in translocating across the nuclear membrane.

Unlike what was observed for the tetra-EGFP–NLS constructs (Fig. 2A and B), we did not find any reduction in the translocation efficiency of H06-IPSE compared with that of H03-IPSE, suggesting that these differences may become apparent only with larger proteins (such as tetra-EGFP) and thus may not be relevant in the context of molecular cross talk between H-IPSE and host cells. In contrast, the NLS H03-IPSE SKAAAKY mutant, despite potentially being able to cross nuclear pores due to its low molecular mass, remained completely excluded from the nucleus (Fig. 5). The H03-IPSE mutant appears to be located in vacuoles or endosome-like structures, mainly around the nucleus, rather than being diffuse in the cytoplasm. This suggests that an intact NLS might be an important feature needed, e.g., for endosomal escape.

The lack of uptake of the Ala mutant shows that the NLS in H-IPSE is monopartite and can be described as being necessary and sufficient; i.e., no nuclear translocation occurs in its absence, and it is the only NLS in the molecule. This is consistent with the lack of a prediction of additional nuclear translocation signals elsewhere in the molecule. These data also demonstrate that the NLS is not required for H-IPSE transport into cells.

### H-IPSE mRNA is expressed in S. haematobium adult females and the egg stage.

**FIG 6:**
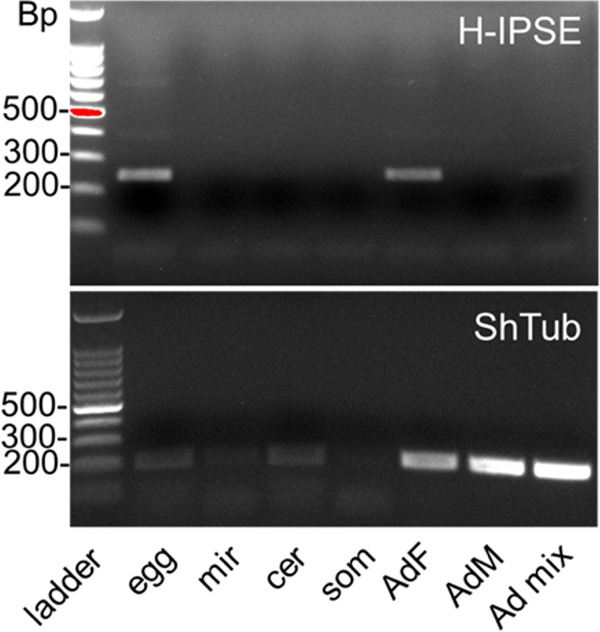
Stage-specific expression of H-IPSE mRNA. Shown are RT-PCR results for H-IPSE obtained from cDNAs prepared by reverse transcription of DNase-treated RNAs isolated at various life stages of S. haematobium. Ladder, 100-bp DNA ladder; egg, S. haematobium egg cDNA; mir, miracidial cDNA; cer, cercarial cDNA; som, *in vitro* mechanically transformed schistosomulum cDNA; AdF, AdM, and Ad mix, mixed cDNAs from female, male, and mixed adult worms, respectively; ShTub, S. haematobium tubulin (control housekeeping gene).

Next, we investigated the expression of H-IPSE across the different life cycle stages, using conventional reverse transcription-PCR (RT-PCR). The results are shown in Fig. 6. RT-PCR data indicated that adult worm cDNA preparations were more contaminated with genomic DNA than were preparations from other life cycles in control experiments; however, DNase treatment completely removed genomic DNA. Using DNase-treated samples, RT-PCR indicated the expression of H-IPSE mRNA in purified eggs and female adult worms and weak expression in mixed-gender adult worms but no expression in cercariae, schistosomula, miracidia, or male adult worms.

Overall, these results are consistent with an expression pattern restricted to the egg stage (since female worms often contain immature eggs) and are similar to those described previously for M-IPSE (7). Interestingly, despite the detection of H-IPSE transcripts in adult female worms, we detected no H-IPSE protein in adult worm antigen (AWA) preparations by Western blotting (Fig. 1B).

## DISCUSSION

The concurrent presence of a CSS and an NLS in the same protein, two apparently contradictory signals in terms of subcellular targeting, is a rare feature. Only 4 of 19 tested algorithms correctly identified the presence of a C-terminal, monopartite NLS in H03-IPSE (cNLSMapper [12], PSORT II [13], NLStradamus [14], and NucPred [15]), while most other tested programs predicted a secretory pathway, some unexpectedly also after the removal of the signal sequence. Thus, a prediction of an NLS by algorithms is still insufficient, and such predictions need to be verified experimentally. At least three properties need to be fulfilled in order to confirm the functionality of an NLS: (i) a functional NLS needs to be able to direct the protein to the nucleus, (ii) the NLS in isolation should also be able to direct heterologous proteins to the nucleus, and, finally, (iii) the mutation of one or several basic amino acids should lead to the loss of translocation or, in the case of a single substitution, at least a weakening of NLS functionality.

Our data confirm that the nuclear localization signals present in H03- and H06-IPSE are functional and essential for the translocation of IPSE into the nuclei of host cells. This is somewhat surprising, as a molecular mass of 40-kDa is well below the known limit for passive diffusion across the nuclear pore complex, which has been described as being “quite larger than 60 kDa” (19). A possible explanation for such behavior could be yet-to-be-characterized interactions with cellular structures or soluble proteins in the cytosol, making the resulting complex too large for passive diffusion into the nucleus.

Kosugi and coauthors described six classes of NLSs with different specificities for the binding grooves of the karyopherin importin-α (17). Based on their classification, the H03-IPSE SKRRRKY sequence would be considered a class I classical NLS, characterized by a stretch of at least four consecutive basic amino acids (either K or R). In contrast, the NLS SKRGRKY in H06-IPSE conforms to a class II classical NLS with the consensus sequence K(K/R)X(K/R), in which one nonbasic amino acid interrupts the adjacency of the basic amino acids found in class I signals, reducing the signal to an interrupted sequence of three basic amino acids. The only difference from the canonical patterns predicted by Kosugi et al. is that both H03- and H06-IPSE appear to possess one additional basic residue in their NLSs. Both classes are predicted to bind to the large major binding pocket of importin-α (17).

Having shown that the NLS in H-IPSE is fully functional, and bearing in mind that the sequence cannot be functional within schistosome eggs themselves, as the N-terminal CSS will target the protein for secretion well before the C-terminal NLS is synthesized, the key question is what the biological function of such a protein might be. The restriction of its expression to a single stage of the parasitic life cycle (the egg stage), which is in line with the restricted expression of the M-IPSE homolog (7), suggests a specialized function needed only during a specific phase of egg embryogenesis or a function needed to govern the very important interactions with host cells and tissues. The former is unlikely due to the secretory nature of the molecule. To further explore this possibility, we first need to summarize the fate of eggs after oviposition by female schistosomes. Newly deposited eggs do not have the complex morphology found in mature eggs; this has been described in detail for S. mansoni by Neill et al. (20), Ashton et al. (21), and Jurberg and coauthors (22). Fewer details are available regarding S. haematobium. Immature eggs are smaller and characterized by the absence of the two envelopes surrounding the miracidia in mature eggs: the outer envelope (Reynolds' layer [RL]) and the inner envelope (von Lichtenberg's layer [vLL]). The RL is enriched with tightly packed rough endoplasmic reticulum structures and is therefore thought to be a major site of protein synthesis. Under an electron microscope, the RL appears richly surrounded by granular materials in a 1-μm-wide space underneath the egg shell (21). Eggs deposited *in vitro* by *ex vivo* worms have been shown to take about a week to fully develop into mature, infective miracidia (23), but development in the host is likely to be more rapid.

The production of M-IPSE in S. mansoni eggs has been clearly shown to occur in the subshell area within the fully formed RL and vLL. This has been demonstrated by immunohistochemical staining with a monoclonal antibody to M-IPSE and by *in situ* hybridization with labeled antisense transcripts of full-length M-IPSE cDNA (7). M-IPSE can also be seen in contact with the tissues around the eggs (confirming that it is secreted by the eggs) (24) and also has been seen inside surrounding host cells (7). Thus, the emerging picture is that immature eggs initially do not produce IPSE but that this protein is produced as eggs mature while migrating through tissues, releasing it into the tissues, where it is able to enter host cells. Inside host cells, it rapidly (in less than half an hour [16]) translocates to the nucleus, where it binds DNA (F. H. Falcone, unpublished data), with yet-to-be-described downstream effects.

It is very clear, however, that in order for the eggs to reach the lumen of the bladder or gut, translocation across host tissues is an event of paramount importance in the life cycle of the parasite. Hence, it can be assumed that molecules secreted exclusively by more mature forms of this life stage may be involved in the egress process and have evolved under high evolutionary pressure.

The first step after oviposition is the escape of eggs from the venules in which they were deposited; *in vitro*, human cells obtained from umbilical venous endothelial cells (HUVECs) rapidly overgrow eggs directly oviposited onto a monolayer (within 4 h), and a similar behavior is seen when eggs are inserted into umbilical veins (25). More recently, de Walick and coauthors demonstrated the deposition of van Willebrand factor and other prothrombic plasma proteins onto the eggshell of S. mansoni (26). However, such steps occur immediately after oviposition; hence, IPSE and other molecules secreted only after a few days of egg maturation cannot play a role in this initial process. Indeed, it was reported previously that this process was slower in mature eggs obtained from infected rodent livers than in freshly deposited eggs, which may depend on the presence of uterine secretions covering the freshly deposited eggs (25). Once the eggs have reached the perivascular tissues, it takes another few days during which they need to cross several robust layers, including the submucosa; the outer muscularis mucosa, consisting of circular and longitudinal muscle; and the inner mucosa, complete with a basal membrane and a very tight epithelial cell layer, before they can reach the lumen: a seemingly impossible journey?

It is well accepted that the granulomatous reaction induced by the eggs plays a key role in this process (27, 28); much of the research in the past focused on the interactions between immune cells and schistosome eggs. In S. mansoni, eggs have been proposed to exploit gut lymphoid structures known as Peyer's patches as a preferential route of egress into the gut lumen (29); however, a comparable route is not available to S. haematobium in the bladder. This leads to the question of whether, in addition to the inflammatory granulomatous response involving immune cells, any direct interactions with nonimmune cells, such as fibroblasts, muscle cells, or epithelial cells, are also involved in facilitating egg translocation. In this context, it is interesting to note that in S. haematobium-infected animals, uroplakins and claudins involved in epithelial tight junction formation are downregulated after exposure of the bladder to eggs (30). Whether this downregulation, which is likely to aid egg egress by disrupting the integrity of the epithelium, is due to the effects of H-IPSE or other egg-derived components remains to be established. The nuclear translocation and direct effects on gene transcription of H-IPSE are currently under active investigation in our laboratories.

Perhaps the most surprising result was the extent to which an exogenously added parasitic molecule was able to enter host cells. This uptake does not appear to be very selective for specific cell types or animal species, as we have seen the uptake of M-IPSE in human Huh-7, U2-OS, and hamster CHO cell lines (16) as well as HUVECs and human monocyte-derived dendritic cells (F. H. Falcone, unpublished data). H-IPSE was taken up by human HTB-9 and Huh-7 cells and monkey Cos-7 cell lines. This raises the question as to whether or which receptors are involved. For M-IPSE, the uptake mechanism has been shown to involve the carbohydrate residues on the protein and C-type lectin receptors such as the mannose receptor, dendritic cell-specific ICAM3-grabbing nonintegrin (DC-SIGN), and a macrophage galactose-type lectin and the mannose receptor (31). Similar receptors have been shown to be involved in the uptake of other schistosome molecules, such as omega-1 (5) and kappa-5 (31). The uptake of a secreted molecule by dendritic cells and macrophages has also been shown for Schistosoma japonicum Sj16 (32), but there is no information regarding the receptors involved in this process; the expression of Sj16 in Escherichia coli used for the previously described experiments, however, suggests that protein glycosylation does not play a role in the uptake of this molecule.

Thus, the secretion of a molecule by a parasitic life stage that is in intimate contact with host tissues, and its subsequent uptake by host cells, may be a more common feature in the host-parasite relationship than hitherto assumed, at least as far as trematodes are concerned. We propose the term “infiltrin” to denote the ability of such molecules to enter host cells in the course of crossing several barriers (the cell membrane, presumably the endosomal membrane, and, in some cases, the nuclear membrane). The archetypal nuclear infiltrins, characterized by the simultaneous presence of a CSS and an NLS, would be M-IPSE and H-IPSE, while the archetypal cytosolic infiltrin would be omega-1 (5). The ability of exogenous polypeptides to enter human cells crossing biological membranes is not a new finding. This was shown for the first time for transactivating transcriptional activator (Tat) of human immunodeficiency virus type 1 (HIV-1) in 1988 (33) and for the 60-amino-acid peptide encoded by the antennapedia gene homeobox in Drosophila melanogaster (34). However, with the exception of our previous report (16), such a principle has not been described for molecules secreted by macroparasites, which are too large to enter host cells. HIV-1 Tat can also carry heterologous proteins across the cell membrane (35), a process now understood to be mediated via a caveolin-dependent uptake route (36). Interestingly, both HIV-1 Tat and Drosophila antennapedia homeobox peptide exhibit DNA binding activities, which are also predicted *in silico* for H-IPSE. Whether H-IPSE has similar properties is also under investigation. We believe that these observations make a compelling case warranting more in-depth studies of parasitic infiltrins and their potential roles as pathogen-derived nuclear transcription factors.

Finally, it needs to be noted that the ability of H-IPSE to enter host cells is not dependent on an intact NLS, as the H03-IPSE NLS mutant as well as the previously described M-IPSE NLS mutants (16) are also able to enter mammalian cells. The same is true for HIV-1 Tat, where the regions responsible for cellular uptake and nuclear translocation are distinct (37, 38). Our data suggest that in the absence of an intact NLS, H-IPSE is able to enter host cells but remains trapped in endosome-like vesicles with a perinuclear distribution. Whether NLS mutants retain their ability to bind host DNA remains to be established.

Taken together, we suggests that nuclear infiltrins, by acting, e.g., as transcription factors, might play a central role in controlling the host-parasite relationship at the molecular level.

## MATERIALS AND METHODS

### Animal experimental protocol.

All animal work was conducted according to relevant U.S. and international guidelines. Specifically, animal experimental protocols were reviewed and approved by the Institutional Animal Care and Use Committee (IACUC) of the Biomedical Research Institute, Rockville, MD. Our IACUC guidelines are in compliance with the U.S. Public Health Service Policy on Humane Care and Use of Laboratory Animals (https://grants.nih.gov/grants/olaw/references/phspol.htm).

### Cloning of IPSE transcripts.

Total RNA was isolated from S. haematobium eggs from the liver of infected hamsters by using TRIzol. Following DNase treatment and inactivation, cDNA was generated from RNA samples by using the Superscript III first-strand cDNA synthesis kit (Invitrogen) with oligo(dT) primers or custom oligo(dT) primers with a 5′-anchoring sequence corresponding to 3′-RACE reverse primers (see Table S1 in the supplemental material). The resulting cDNA was then amplified by either targeted 5′ and 3′ primers designed from predicted H-IPSE transcripts or nested 3′-RACE PCR with a 5′ primer targeted to conserved 5′ elements in IPSE by using Platinum *Taq* Supermix (Invitrogen). Gel-purified PCR fragments were then treated with *Taq* polymerase to facilitate cloning into the pCR 2.1-TOPO vector included with the Topo TA cloning kit (Invitrogen), and all positive colonies from the blue/white assay were sequenced.

### Recombinant protein expression in E. coli BL21 Star(DE3).

The H06-IPSE protein-coding sequence lacking the N-terminal signal sequence was cloned into the pET-100D Topo expression vector (Invitrogen) and transformed into E. coli BL21 Star(DE3). One-liter cell cultures were grown to an optical density (OD) of 0.6 under ampicillin selection (100 μg/ml) and were induced with 1 mM isopropyl-β-d-thiogalactopyranoside (IPTG) for 3 h before harvest. Cell pellets were then suspended in 50 ml of lysis buffer (6 M guanidine hydrochloride, 10 mM imidazole, 20 mM sodium phosphate, and 500 mM NaCl at pH 7.8) and treated with EDTA-free protease inhibitor tablets (Pierce). Cells were lysed with three freeze-thaw cycles and sonicated on ice. Nickel-nitrilotriacetic acid (NTA) resin purifications were conducted with binding buffer containing 8 M urea, 10 mM imidazole, and phosphate-buffered saline (PBS) at pH 7.4; wash buffer consisting of 8 M urea, 25 mM imidazole, and PBS at pH 7.4; and elution buffer containing 8 M urea, 300 mM imidazole, and PBS at pH 7.4. This solution was successively dialyzed against PBS (pH 7.4) solutions containing 4.0 M, 2.0 M, and 1.0 M urea over 3 days before being dialyzed overnight against PBS (see Fig. S4A in the supplemental material). Refolded protein was concentrated to <0.50 mg/ml in a 3.0-kDa-cutoff Centricon centrifugal concentrator (EMD Millipore).

### Generation of polyclonal IPSE antibodies.

Recombinant bacterially derived H06-IPSE protein (obtained as described above) was used to immunize rabbits 4 times over the course of 8 weeks (ProMab Biotechnologies Inc.) (see Fig. S1B in the supplemental material). Antibody was precipitated from sera by using ammonium sulfate and suspended in PBS with 0.03% sodium azide prior to use.

### Cloning of H-IPSE into the pTT5 expression vector.

To facilitate mammalian expression, codon-optimized synthetic IPSE vectors were generated for H03-IPSE, H06-IPSE, and M-IPSE (GeneArt; Invitrogen). The IPSE variants were PCR amplified from these synthetic constructs. A second insert containing the human vascular endothelial growth factor (VEGF) signal sequence, an 8× His tag, and a tobacco etch virus (TEV) cleavage site was also amplified. These two fragments were inserted into an EcoRI/NheI-digested pTTVH8G vector (licensed from the Canadian Research Council [39]) by Gibson assembly. Subsequently, during vector optimization, the full IPSE expression cassette with the N-terminal signal sequence, tag, and cleavage site was transferred from the pTTVH8G vector to the pTT5 vector by conventional restriction cloning using EcoRI and NotI. The H06-IPSE SKAAAKY NLS mutant was cloned by site-directed mutagenesis of H06-pTT5 vectors by using Phusion High Fidelity PCR master mix (Invitrogen), followed by DpnI digestion for 1 h.

### RT-PCR and stage-specific expression of H-IPSE.

Total RNA was isolated from the following S. haematobium life cycle stages (obtained from the NIAID Schistosomiasis Resource Center for distribution through BEI Resources, NIAID, NIH), using the RNAzol kit (Molecular Research Center) according to the manufacturer's instructions: purified eggs, retrieved from the liver of S. haematobium-infected hamsters; miracidia; cercariae; schistosomula; adult females; adult males; and mixed-sex adult worms. Contaminating genomic DNA was removed by DNase treatment using Turbo DNase (Invitrogen Ambion, USA) and chemical DNase inactivation, according to the manufacturer's instructions. After the removal of genomic DNA contaminants, cDNA was obtained by reverse transcription using an iScript cDNA synthesis kit (Bio-Rad, USA) according to the manufacturer's directions. After reverse transcription, RNA sample concentrations were measured by using a NanoDrop ND-1000 spectrophotometer (Thermo Fisher Scientific) and adjusted to 500 ng/μl for all samples by using molecular-grade water. PCR was performed on a Bio-Rad CFX Connect thermocycler under the following cycling conditions: an initial denaturation step (2 min at 94°C) followed by 35 cycles of denaturation (30 s at 94°C), annealing (45 s at 56°C), and extension (1 min at 72°C) and a final extension step (5 min at 72°C). The polymerase used was TaKaRa *Taq* polymerase, using 2 μl 10× polymerase buffer, 2 μl 10 μM deoxynucleoside triphosphates (dNTPs), 1 μl each of forward (5′-GCTCACTCTCACCACCATG-3′) and reverse (5′-TCCTTCGACGTTTCGATTCAC-3′) primers, 2 μl of the cDNA template, and 11.5 μl molecular-grade water in a 20-μl total volume. PCR mixtures were then subjected to electrophoretic separation on 1% agarose containing 0.5 μg/ml ethidium bromide in 0.5× Tris-borate-EDTA (TBE) buffer. A 100-bp DNA ladder (Promega, USA) was used for sizing of the amplicon. Gels were imaged by using a GelDoc XR molecular imager (Bio-Rad, USA) and saved as .tif files. The oligonucleotide primers used span an intron, discriminating cDNA (295 bp) from genomic DNA (402 bp).

### Recombinant protein expression using HEK293-6E cells.

H03-IPSE and H06-IPSE were recombinantly expressed for uptake and microscopy experiments using the pTT5 HEK293-6E expression platform (National Research Council Canada file 11565) licensed from the Canadian Research Council (39). A large-scale gene expression workflow was developed by using 2-liter vented shaking flasks. Cells were cultivated in suspension in an incubator at 37°C in a 5% CO_2_ humidified atmosphere under constant shaking at a rate of 120 rpm. The medium consisted of Freestyle F17 medium (Gibco, Rockville, MD) supplemented with 0.1%, (wt/vol) Kolliphor P-188 (Sigma-Aldrich) with 4 mM l-glutamine and 25 μg/ml G418 (Thermo Fisher Scientific). Freestyle 293 medium (Gibco, Rockville, MD) was used interchangeably with F17 medium without glutamine supplementation.

For transient transfection, 500 ml of the cell suspension was mixed with 12.5 ml of medium containing the plasmid DNA and another 12.5 ml of medium for resuspension of linear 25-kDa polyethylenimine (PEI) (PolyPlus). The final DNA amount for each pTT construct was 0.5 mg, and this was with mixed 1.5 mg of PEI (3:1 PEI/DNA ratio) and incubated for 3 min at room temperature. The resultant complex was then added to the cells. A total of 2.5 ml of 20% (wt/vol) tryptone N1 (TN1; TekniScience Inc., Canada) was added 24 h after transfection.

Wild-type (WT) or mutant H-IPSE proteins, secreted into HEK293-6E serum-free cell culture medium, were harvested 7 days after transfection, followed by protein purification. This supernatant was centrifuged at 2,800 × *g* for 10 min (4°C), followed by 0.22-μm filtration to remove cell debris and aggregates, and then purified by IMAC using Talon Superflow cobalt affinity resin (GE Healthcare, Freiburg, Germany) or Ni-NTA agarose (Qiagen). For cobalt resin purification, binding buffer consisted of 50 mM sodium phosphate and 300 mM NaCl (pH 7.4), and wash buffer contained 50 mM sodium phosphate, 300 mM NaCl, and 5 mM imidazole (pH 7.4), while elution buffer consisted of 50 mM sodium phosphate, 300 mM NaCl, and 150 mM imidazole (pH 7.4). Nickel resin purifications were conducted with binding buffer containing PBS (pH 7.4), wash buffer consisting of PBS and 10 mM imidazole at pH 7.4, and elution buffer containing PBS and 300 mM imidazole at pH 7.4.

### SDS-PAGE, Coomassie staining, and Western blotting.

Fifteen-microliter aliquots of the eluted purified protein fractions were separated by SDS-PAGE using 4-20% gradient gels (Mini-Protean TGX Precast gels; Bio-Rad), as recommended by the manufacturer. The gels were then incubated for half an hour in Instant Blue (Expedeon, Harston, UK) for Coomassie staining, followed by washing in deionized water. For Western blotting, the gradient TGX gels were transferred to 0.2-μm nitrocellulose membranes by using the Trans-Blot Turbo transfer system according to the manufacturer's protocols (Bio-Rad). The membranes were blocked with blocking buffer (5% [wt/vol] dried skim milk, 0.01% [vol/vol] Tween 20, and Tris-buffered saline [TBS]) with shaking for 1 h at room temperature. Next, membranes were incubated with primary mouse anti-His antibody (GE Healthcare) as the primary antibody, which was diluted 1:5,000, at 4°C overnight, followed by three washes in a TBS solution containing 1% Tween for 10 min each. The membranes were then incubated with anti-mouse IgG (whole molecule) and horseradish peroxidase (HRP)-conjugated antibody (Sigma-Aldrich, UK) as a secondary antibody (1:4,000) for 1 h at room temperature, followed by washing as described above. Membranes were imaged by using a Fuji LAS4000 imager with chemiluminescence-luminol reagent (3 μl of 30% H_2_O_2_, 0.1 mM Tris-HCl [pH 8], 2.5 mM luminol, and 400 μM coumaric acid). For the detection of the native IPSE protein in adult worm extracts and egg-derived samples, approximately 40 μg of parasite-derived material was loaded per well, as determined by the *A*_280_ for each sample, using a spectrophotometer. These samples were run on 4 to 20% ExpressPlus PAGE gels (GenScript) according to the manufacturer's instructions and stained with Coomassie brilliant blue G250 (Bio-Rad). For Western blotting, gels were transferred to a 0.22-μm polyvinylidene difluoride (PDVF) membrane after preactivation with methanol. The membranes were blocked with blocking buffer (5% [wt/vol] dried skim milk, 0.01% [vol/vol] Tween 20, and PBS) with shaking for 1 h at room temperature. Next, blots were incubated with polyclonal anti-H06-IPSE rabbit antibodies at a 1:500 dilution and stained overnight at 4°C. After three washes in PBS-Tween (PBS-T), blots were then developed with polyclonal HRP-conjugated goat anti-rabbit secondary antibody (EMD Millipore) at a dilution of 1:5,000. The gels were washed three additional times in PBS-T and developed with the SignalFire ECL reagent (Cell Signaling).

### Cloning of the predicted NLS and mutants into pTetra-EGFP.

The predicted NLS for each protein was subcloned into the tetra-EGFP vector, which carries a kanamycin resistance gene (40). This vector encodes four EGFP repeats with a multiple-cloning site inserted between the third and fourth EGFP sequences. The nucleotides encoding the predicted NLSs in H03-IPSE and H06-IPSE, as well as the predicted NLS mutant and the canonical simian virus 40 (SV40) NLS, were inserted into pTetra-EGFP by using oligonucleotide primers and specific restriction enzymes (see Table S1 in the supplemental material). This leads to a construct that codes for a tetra-EFGP fusion protein of approximately 113 kDa that, due to its large size, is completely excluded from the nucleus in the absence of a functional NLS. Initially, 5′-phosphorylated pairs of matching oligonucleotides were designed to encode putative NLSs containing GATC overhangs by using 1 μl of each oligonucleotide (100 μM) mixed with 98 μl of a solution containing 10 mM Tris-HCl and 1 mM EDTA (pH 8.0), followed by denaturation at 95°C for 7 min and then at 5°C for 3 min. Next, the double-stranded oligonucleotides and the pTetra-EGFP vector were digested with the restriction enzyme BglII (New England BioLabs), according to the manufacturer's protocols. The ends of the linearized pTetra-EGFP vector were dephosphorylated with Antarctic phosphatase (New England BioLabs) to avoid religation to itself, according to the manufacturer's instructions. Ligation was performed by using 1 μl vector, 3 μl insert, 1 μl 10× buffer-T4 ligase, 4 μl molecular-biology-grade water, and 1 U T4 DNA ligase (Promega) in a 10-μl reaction mixture, and the mixture was incubated overnight at 16°C. DNA sequencing (Source BioScience, UK) using T7 primers determined successful insertion in the correct orientation.

### HTB-9 cell culture and transfection.

The human bladder cancer cell line HTB-9 (ATCC 5637) was grown in T75 flasks (Sarstedt, Germany) at 37°C in a humidified 5% CO_2_ incubator with Eagle minimum essential medium (MEM; Sigma-Aldrich) supplemented with 5% heat-inactivated fetal bovine serum (FBS; Gibco), 2 mM l-glutamine, 100 U/ml penicillin, and 100 μg/ml streptomycin (Sigma-Aldrich, UK). Transient transfections of HTB-9 cells were performed by using the X-tremeGENE9 DNA transfection reagent (Roche Applied Science, Germany) according to the manufacturer's protocols. Cells were plated onto 5 mg/ml rat tail collagen I-coated glass coverslips (15-mm diameter and 0.13- to 0.16-mm thickness; Invitrogen, UK) in 6-well plates and transfected with the different tetra-EGFP plasmids at 60 to 70% confluence.

### Cell fixation and fluorescence microscopy.

One day after transfection, the cells were washed with Dulbecco's phosphate-buffered saline (DPBS; Gibco) and fixed at room temperature for 10 to 15 min in 4% paraformaldehyde. The cells were then washed three times with DPBS and incubated with 0.5 g/ml Hoechst 33342 stain (Sigma-Aldrich) at room temperature for 8 to 15 min, before being washed again three times with DPBS. Slides were mounted with mounting medium (Sigma-Aldrich). Transfected cells were visualized by fluorescence microscopy (Evos fl; Advanced Microscopy Group, USA) or confocal microscopy (LSM510 Meta; Zeiss, Germany) and analyzed by using Zeiss LSM Image Browser software (version 4.2.0.121).

### Cellular uptake of H03-IPSE.

HTB9 cells were seeded onto Lab-Tek 8-well chambered cover glass (Nalgene Nunc International) at a density of 5 × 10^5^ cells in order to achieve 50 to 60% confluence after 24 h. Cells were then incubated with 15 to 0.40 nM recombinant proteins (WT or mutant H03-IPSE protein) in serum-free internalization medium (HEPES-buffered Ham's) F-12 medium (Sigma-Aldrich, UK) containing 10 mM NaHCO_3_ and 2 mg/ml bovine serum albumin (fraction V; Biomol GmbH, Germany), followed by fixation at room temperature for 10 to 15 min in a 4% paraformaldehyde solution. The cells were then washed 4 to 5 times with DPBS, incubated with 0.5 μg/ml Hoechst 33342 or 5 μM DRAQ5 nuclear stain (Thermo Fisher Scientific) for 15 min, and permeabilized with 0.2% Triton X-100 in DPBS for 10 min. The cells were washed 4 to 5 times with DPBS and incubated at room temperature for 30 min with mouse anti-His antibody (GE Healthcare) diluted 1:5,000. The cells were then washed three times and labeled with Alexa Fluor 555-conjugated goat anti-mouse IgG(H+L) secondary antibody (Molecular Probes) diluted 1:500 by incubation at room temperature for 30 min, followed by three final washes with DPBS.

### Accession number(s).

The nucleotide sequence data have been deposited in GenBank under the following accession numbers: for H01-IPSE, MG012891; for H03-IPSE, MG012892; for H04-IPSE, MG012893; for H06-IPSE, MG012894; for H07-IPSE, MG012895; for H08-IPSE, MG012896; for H09-IPSE, MG012897; and for H10-IPSE, MG012898.

## Supplementary Material

Supplemental material
