# Supplementary material for: H-IPSE Is a Pathogen-Secreted Host Nucleus-Infiltrating Protein (Infiltrin) Expressed Exclusively by the Schistosoma haematobium Egg Stage
Source: Infect Immun. 2017 Nov 17;85(12):e00301-17. doi: 10.1128/IAI.00301-17 (PMC5695104; doi:10.1128/IAI.00301-17)
Supplement: Supplemental material [file supp_85_12_e00301-17__index.html]

Supplemental material 

# H-IPSE Is a Pathogen-Secreted Host Nucleus-Infiltrating Protein (Infiltrin) Expressed Exclusively by the Schistosoma haematobium Egg Stage

## Supplemental material

- Supplemental file 1 -

  Fig. S1. Geneious global alignment with free end gaps and default alignment. Fig. S2. Matrix showing the relative nucleotide identities of the indicated sequences. Fig. S3. FASTA-formatted sequences used for alignments. Fig. S4. Generation of polyclonal anti-H06-IPSE antibodies. Fig. S5. Mammal-derived IPSE: 4–20% gradient SDS-PAGE of IPSE variants expressed in HEK-6E cells and purified with Ni-NTA or TALON affinity resins stained with Coomassie R250. Table S1. Oligonucleotide sequences used in this study.

  PDF, 1.9M
