# Supplementary material for: H-IPSE Is a Pathogen-Secreted Host Nucleus-Infiltrating Protein (Infiltrin) Expressed Exclusively by the Schistosoma haematobium Egg Stage
Source: Infect Immun. 2017 Nov 17;85(12):e00301-17. doi: 10.1128/IAI.00301-17 (PMC5695104; doi:10.1128/IAI.00301-17)
Supplement: Supplemental material [file IAI.00301-17_zii999092214s1.pdf]

SUPPLEMENTARY INFORMATION TO

Manuscript IAI#00301-17

H-IPSE is a pathogen-secreted host nucleus infiltrating protein (infiltrin) expressed exclusively by the *Schistosoma haematobium* egg stage

Luke F. Pennington[a$], Abdulaziz Alouffi[b$] Evaristus C. Mbanefo[c], Debalina Ray[d], David M. Heery[e], Ted Jardetzky[a], Michael H. Hsieh[c,f] and F. H. Falcone[b]

[a]Stanford University School of Medicine, Stanford, CA, USA;
[b]School of Pharmacy, Division of Molecular Therapeutics and Formulation, University of Nottingham, Nottingham, UK;
[c] Biomedical Research Institute, Rockville, MD, USA
[d]Department of Pathology, University of California, San Francisco, CA,USA
[e]School of Pharmacy, Division of Biomolecular Science and Medicinal Chemistry, University of Nottingham, Nottingham, UK
[f]Department of Urology, The George Washington University, Washington, D.C., USA

[$] Equal contributors

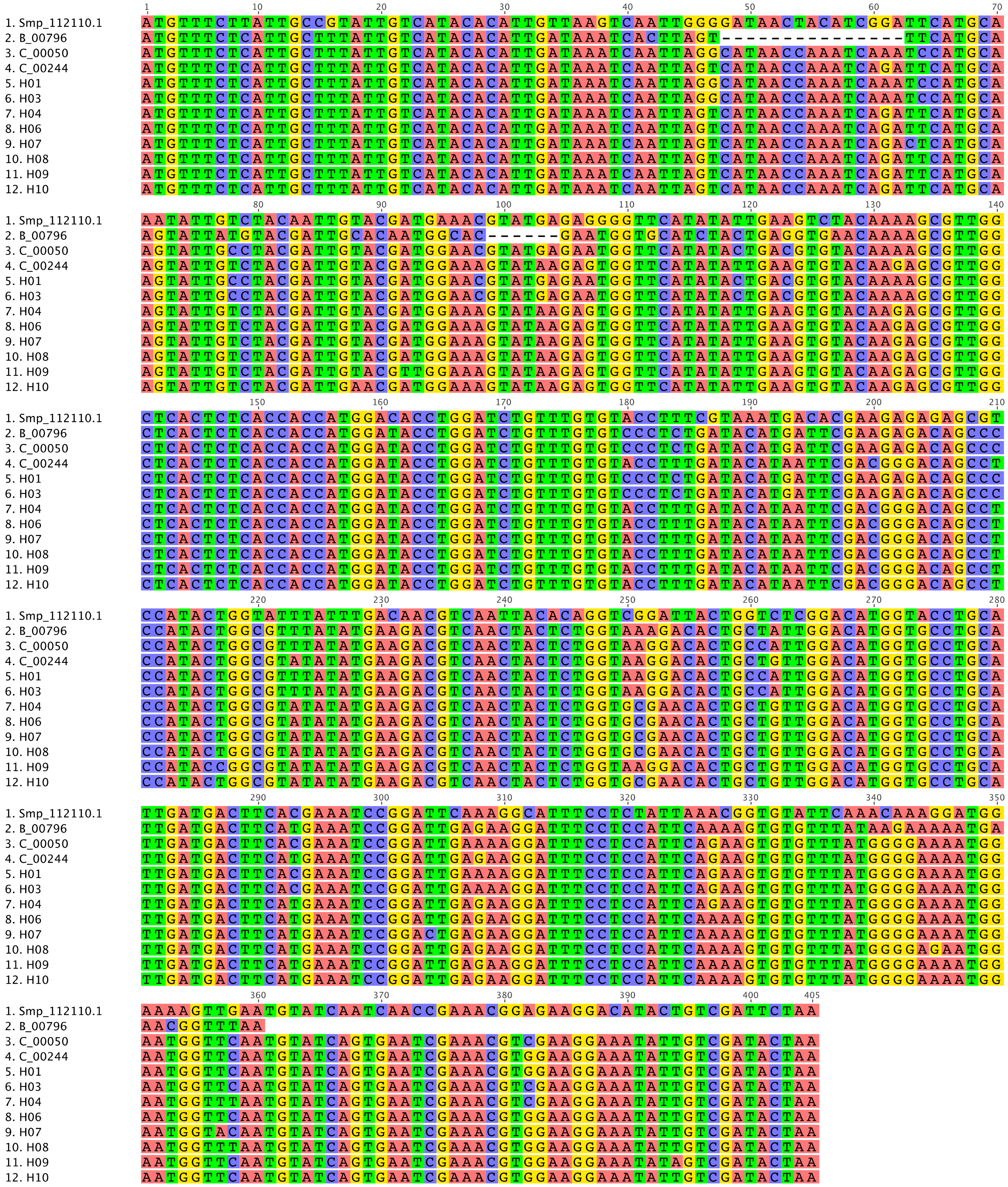

Figure S1: A Geneious global alignment with free end gaps and default alignment parameters (65% similarity (5.0/-4.0) cost matrix, 12 gap open penalty, 3 gap extension penalty, 2 refinement iterations) was performed using indicated sequences (Geneious v7.1.4).

	Smp_112110.1	B_00796	C_00050	C_00244	H01	H03	H04	H06	H07	H08	H09	H10
**Smp_112110.1**		73.9	80.7	80.7	80.7	80.7	81	81.2	80.5	81.5	80.2	81
**B_00796**	73.9		87.5	85.8	87.5	87.5	85.3	85.3	84.4	85.3	85.6	85.3
**C_00050**	80.7	87.5		94.6	99.8	100	93.8	93.6	92.8	93.1	93.6	93.3
**C_00244**	80.7	85.8	94.6		94.8	94.6	98.8	99	98.3	98.5	99	98.8
**H01**	80.7	87.5	99.8	94.8		99.8	93.6	93.8	93.1	93.3	93.8	93.6
**H03**	80.7	87.5	100	94.6	99.8		93.8	93.6	92.8	93.1	93.6	93.3
**H04**	81	85.3	93.8	98.8	93.6	93.8		99.3	98.5	99.3	97.8	99
**H06**	81.2	85.3	93.6	99	93.8	93.6	99.3		99.3	99.5	98.5	99.8
**H07**	80.5	84.4	92.8	98.3	93.1	92.8	98.5	99.3		98.8	97.8	99
**H08**	81.5	85.3	93.1	98.5	93.3	93.1	99.3	99.5	98.8		98	99.3
**H09**	80.2	85.6	93.6	99	93.8	93.6	97.8	98.5	97.8	98		98.3
**H10**	81	85.3	93.3	98.8	93.6	93.3	99	99.8	99	99.3	98.3	

Figure S2: A matrix showing the relative nucleotide identity of the indicated sequences.

```
>Smp_112110.1
ATTGCCGTATTGTCATACACATTGTTAAGTCAATTGGGGATAACTACATCGGATTCATGCAAATATTGTCTACAATTG
TACGATGAAACGTATGAGAGGGGTTCATATATTGAAGTCTACAAAAGCGTTGGCTCACTCTCACCACCATGGACACCT
GGATCTGTTTGTGTACCTTTCGTAAATGACACGAAGAGAGAGCGTCCATACTGGTATTTATTTGACAACGTCAATTAC
ACAGGTCGGATTACTGGTCTCGGACATGGTACCTGCATTGATGACTTCACGAAATCCGGATTCAAAGGCATTTCCTCT
ATTAAACGGTGTATTCAAACAAAGGATGGAAAAGTTGAATGTATCAATCAACCGAAACGGAGAAGGACATACTGTCGA
TTCTAA

>C_00244
ATTGCTTTATTGTCATACACATTGATAAATCAATTAGTCATAACCAAATCAGATTCATGCAAGTATTGTCTACGATTG
TACGATGGAAAGTATAAGAGTGGTTCATATATTGAAGTGTACAAGAGCGTTGGCTCACTCTCACCACCATGGATACCT
GGATCTGTTTGTGTACCTTTGATACATAATTCGACGGGACAGCCTCCATACTGGCGTATATATGAAGACGTCAACTAC
TCTGGTAAGGACACTGCTGTTGGACATGGTGCCTGCATTGATGACTTCATGAAATCCGGATTGAGAAGGATTTCCTCC
ATTCAGAAGTGTGTTTATGGGGAAAATGGAATGGTTCAATGTATCAGTGAATCGAAACGTGGAAGGAAATATTGTCGA
TACTAA

>C_00050
ATTGCTTTATTGTCATACACATTGATAAATCAATTAGGCATAACCAAATCAAATCCATGCAAGTATTGCCTACGATTG
TACGATGGAACGTATGAGAATGGTTCATATACTGACGTGTACAAAAGCGTTGGCTCACTCTCACCACCATGGATACCT
GGATCTGTTTGTGTCCCTCTGATACATGATTCGAAGAGACAGCCCCCATACTGGCGTTTATATGAAGACGTCAACTAC
TCTGGTAAGGACACTGCCATTGGACATGGTGCCTGCATTGATGACTTCACGAAATCCGGATTGAAAAGGATTTCCTCC
ATTCAGAAGTGTGTTTATGGGGAAAATGGAATGGTTCAATGTATCAGTGAATCGAAACGTCGAAGGAAATATTGTCGA
TACTAA

>B_00796
ATTGCTTTATTGTCATACACATTGATAAATCACTTAGTTTCATGCAAGTATTATGTACGATTGCACAATGGCACGAAT
GGTGCATCTACTGAGGTGAACAAAAGCGTTGGCTCACTCTCACCACCATGGATACCTGGATCTGTTTGTGTCCCTCTG
ATACATGATTCGAAGAGACAGCCCCCATACTGGCGTTTATATGAAGACGTCAACTACTCTGGTAAAGACACTGCTATT
GGACATGGTGCCTGCATTGATGACTTCATGAAATCCGGATTGAGAAGGATTTCCTCCATTCAAAAGTGTGTTTATAAG
AAAAATGAAACGGTTTAA

>H1
ATTGCTTTATTGTCATACACATTGATAAATCAATTAGGCATAACCAAATCAAATCCATGCAAGTATTGCCTACGATTG
TACGATGGAACGTATGAGAATGGTTCATATACTGACGTGTACAAAAGCGTTGGCTCACTCTCACCACCATGGATACCT
GGATCTGTTTGTGTCCCTCTGATACATGATTCGAAGAGACAGCCCCCATACTGGCGTTTATATGAAGACGTCAACTAC
TCTGGTAAGGACACTGCCATTGGACATGGTGCCTGCATTGATGACTTCACGAAATCCGGATTGAAAAGGATTTCCTCC
ATTCAGAAGTGTGTTTATGGGGAAAATGGAATGGTTCAATGTATCAGTGAATCGAAACGTGGAAGGAAATATTGTCGA
TACTAA

>H3
```

```
ATTGCTTTATTGTCATACACATTGATAAATCAATTAGGCATAACCAAATCAAATCCATGCAAGTATTGCCTACGATTG
TACGATGGAACGTATGAGAATGGTTCATATACTGACGTGTACAAAAGCGTTGGCTCACTCTCACCACCATGGATACCT
GGATCTGTTTGTGTCCCTCTGATACATGATTCGAAGAGACAGCCCCCATACTGGCGTTTATATGAAGACGTCAACTAC
TCTGGTAAGGACACTGCCATTGGACATGGTGCCTGCATTGATGACTTCACGAAATCCGGATTGAAAAGGATTTCCTCC
ATTCAGAAGTGTGTTTATGGGGAAAATGGAATGGTTCAATGTATCAGTGAATCGAAACGTCGAAGGAAATATTGTCGA
TACTAA

>H4
ATTGCTTTATTGTCATACACATTGATAAATCAATTAGTCATAACCAAATCAGATTCATGCAAGTATTGTCTACGATTG
TACGATGGAAAGTATAAGAGTGGTTCATATATTGAAGTGTACAAGAGCGTTGGCTCACTCTCACCACCATGGATACCT
GGATCTGTTTGTGTACCTTTGATACATAATTCGACGGGACAGCCTCCATACTGGCGTATATATGAAGACGTCAACTAC
TCTGGTGCGAACACTGCTGTTGGACATGGTGCCTGCATTGATGACTTCATGAAATCCGGATTGAGAAGGATTTCCTCC
ATTCAGAAGTGTGTTTATGGGGAAAATGGAATGGTTTAATGTATCAGTGAATCGAAACGTCGAAGGAAATATTGTCGA
TACTAA

>H6
ATTGCTTTATTGTCATACACATTGATAAATCAATTAGTCATAACCAAATCAGATTCATGCAAGTATTGTCTACGATTG
TACGATGGAAAGTATAAGAGTGGTTCATATATTGAAGTGTACAAGAGCGTTGGCTCACTCTCACCACCATGGATACCT
GGATCTGTTTGTGTACCTTTGATACATAATTCGACGGGACAGCCTCCATACTGGCGTATATATGAAGACGTCAACTAC
TCTGGTGCGAACACTGCTGTTGGACATGGTGCCTGCATTGATGACTTCATGAAATCCGGATTGAGAAGGATTTCCTCC
ATTCAAAAGTGTGTTTATGGGGAAAATGGAATGGTTCAATGTATCAGTGAATCGAAACGTGGAAGGAAATATTGTCGA
TACTAA

>H7
ATTGCTTTATTGTCATACACATTGATAAATCAATTAGTCATAACCAAATCAGACTCATGCAAGTATTGTCTACGATTG
TACGATGGAAAGTATAAGAGTGGTTCATATATTGAAGTGTACAAGAGCGTTGGCTCACTCTCACCACCATGGATACCT
GGATCTGTTTGTGTACCTTTGATACATAATTCGACGGGACAGCCTCCATACTGGCGTATATATGAAGACGTCAACTAC
TCTGGTGCGAACACTGCTGTTGGACATGGTGCCTGCATTGATGACTTCATGAAATCCGGACTGAGAAGGATTTCCTCC
ATTCAAAAGTGTGTTTATGGGGAAAATGGAATGGTACAATGTATCAGTGAATCGAAACGTGGAAGGAAATATTGTCGA
TACTAA

>H8
ATTGCTTTATTGTCATACACATTGATAAATCAATTAGTCATAACCAAATCAGATTCATGCAAGTATTGTCTACGATTG
TACGATGGAAAGTATAAGAGTGGTTCATATATTGAAGTGTACAAGAGCGTTGGCTCACTCTCACCACCATGGATACCT
GGATCTGTTTGTGTACCTTTGATACATAATTCGACGGGACAGCCTCCATACTGGCGTATATATGAAGACGTCAACTAC
TCTGGTGCGAACACTGCTGTTGGACATGGTGCCTGCATTGATGACTTCATGAAATCCGGATTGAGAAGGATTTCCTCC
ATTCAAAAGTGTGTTTATGGGGAGAATGGAATGGTTTAATGTATCAGTGAATCGAAACGTGGAAGGAAATATTGTCGA
TACTAA

>H9
ATTGCTTTATTGTCATACACATTGATAAATCAATTAGTCATAACCAAATCAGATTCATGCAAGTATTGTCTACGATTG
TACGTTGGAAAGTATAAGAGTGGTTCATATATTGAAGTGTACAAGAGCGTTGGCTCACTCTCACCACCATGGATACCT
GGATCTGTTTGTGTACCTTTGATACATAATTCGACGGGACAGCCTCCATACCGGCGTATATATGAAGACGTCAACTAC
TCTGGTAAGGACACTGCTGTTGGACATGGTGCCTGCATTGATGACTTCATGAAATCCGGATTGAGAAGGATTTCCTCC
ATTCAAAAGTGTGTTTATGGGGAAAATGGAATGGTTCAATGTATCAGTGAATCGAAACGTGGAAGGAAATATAGTCGA
TACTAA

>H10
ATTGCTTTATTGTCATACACATTGATAAATCAATTAGTCATAACCAAATCAGATTCATGCAAGTATTGTCTACGATTG
AACGATGGAAAGTATAAGAGTGGTTCATATATTGAAGTGTACAAGAGCGTTGGCTCACTCTCACCACCATGGATACCT
GGATCTGTTTGTGTACCTTTGATACATAATTCGACGGGACAGCCTCCATACTGGCGTATATATGAAGACGTCAACTAC
TCTGGTGCGAACACTGCTGTTGGACATGGTGCCTGCATTGATGACTTCATGAAATCCGGATTGAGAAGGATTTCCTCC
ATTCAAAAGTGTGTTTATGGGGAAAATGGAATGGTTCAATGTATCAGTGAATCGAAACGTGGAAGGAAATATTGTCGA
TACTAA
```

Figure S3: FASTA-formatted sequences used for alignments

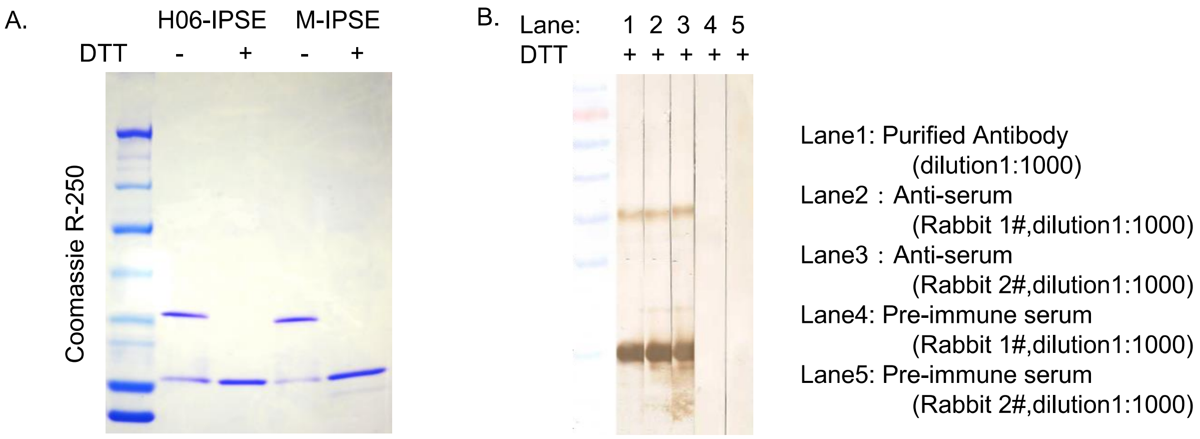

Figure S4: Generation of polyclonal anti-H06-IPSE antibodies: A.) 4-20% gradient SDS-PAGE electrophoresis of refolded H06-IPSE and M-IPSE stained with Coomassie R250. H06-IPSE protein was supplied as immunogen to ProMab Biotechnologies for the generation of anti-IPSE polyclonal rabbit antibodies. B.) A western blot H06-IPSE under reducing conditions with anti-serum and purified antibody from immunized rabbits before and after IPSE injection.

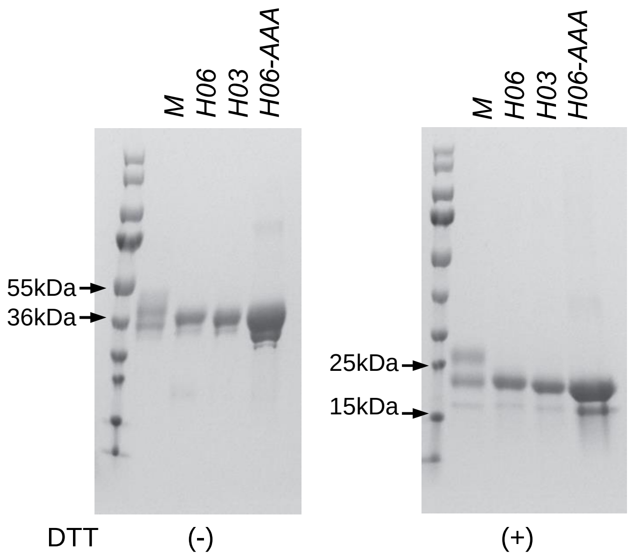

Figure S5: Mammalian derived IPSE: 4-20% gradient SDS-PAGE electrophoresis of IPSE variants expressed in HEK-6E cells and purified with Ni-NTA or TALON affinity resins stained with Coomassie R250. Proteins exhibited the expected homodimeric (DTT negative) and monomeric (DTT positive) molecular weights corresponding to differential glycosylation at the multple potential N-linked glycosylation sites.

Table T1

Mutant/wt	*Purpose*<br>Sequence (5'→3')
	Construction pTT5 expression constructs
M-IPSE for	GGCGGAGAGAACCTGTATTTTCAGGGCGGATCCGAC AGCTGCAAGTACTGCCTGCAG
M-IPSE rev	AGCGGCCGCGGTACCAAGCTAGTCATTACTAGAACC GGCAGTAGGTCCTCCGCCGCTTGG
H03-IPSE for	GGCGGAGAGAACCTGTATTTTCAGGGCGGATCCAAC CCCTGCAAGTACTGCCTGCG
H03-IPSE rev	GCGGCCGCGGTACCAAGCTAGTCATTACTAGTACCG GCAGTACTTTCTCCGCCGCTTGC
H06-IPSE for	GGCGGAGAGAACCTGTATTTTCAGGGCGGATCCAAG AGCGACAGCTGCAAGTACTG
H06-IPSE rev	AGCGGCCGCGGTACCAAGCTAGTCATTACTAGTACC GGCAGTACTTTCTGCCCCGCTTGC
VEGF-SS-8xHIS-TEV for	CCAGGTCCAAGTTTAAACGGATCTCTAGCG
VEGF-SS-8xHIS-TEV rev	GGATCCGCCCTGAAAATACAGGTTCTCTCCGCC
H06-SK**AAA**KY for	GAGAGCAAGGCCGCTGCCAAGTACTGCCGGTACTAG TAATGACTAGC
H06-SK**AAA**KY rev	GCAGTACTTGGCAGCGGCCTTGCTCTCGCTGATGCA CTGCACCATGCC
	Cloning IPSE transcripts
H-IPSE for	ATGTTTCTCATTGCTTTATTGTCATACAC
H-IPSE rev	ACACAAACAGATCCAGGTATCC
H-IPSE RACE for.1	CGATGTTTCTCATTGCTTTATTGTC
H-IPSE RACE (nested) for.2	GTTTCTCATTGCTTTATTGTCATAC
Anchor Insert 3' RACE rev.1	GTCATCGGCTTAGCATTG
Anchor Insert 3' RACE (nested) rev.2	CTTAGCATTGATGTCTCGC
	Tetra-EGFP- NLS mutagenic oligonucleotides
PKKKRKV–For (SV40 pos control)	***GATCT**CCGAAGAAGAAGAGGAAGGTA**A**
PKKKRKV –Rev	***GATCT**TACCTTCCTCTTCTTCTTCGG**A**
SKRGRKY-For (H06-IPSE WT)	***GATCT**AGCAAGAGGGGAAGGAAGTAC**A**
SKRGRKY-Rev	***GATCT**GTACTTCCTTCCCCTCTTGCT**A**
SKR**R**RKY-For (H03-IPSE WT)	***GATCT**AGCAAGAGGAGG**AGG**AAGTAC**A**
SKR**R**RKY-Rev	***GATCT**GTACTTCCTCCT**CCT**CTTGCT**A**
S**AA**G**AA**Y-For (H06 NLS mut)	***GATCT**AGC**GCAGCA**GGA**GCAGCA**TAC**A**
S**AA**G**AA**Y-Rev	***GATCT**GTA**TGCTGC**TCC**TGCTGC**GCT**A**

Table S1. Oligonucleotide sequences used in this study. Key: Bold, underlined: restriction sites; Bold, blue: mutation introduced. * denotes 5'-phosphorylation.
